# Supplementary material for: MELD-accelerated molecular dynamics help determine amyloid fibril structures
Source: Commun Biol. 2021 Aug 5;4:942. doi: 10.1038/s42003-021-02461-y (PMC8342454; doi:10.1038/s42003-021-02461-y)
Supplement: Supplementary file 2 — Supplementary Information [file 42003_2021_2461_MOESM2_ESM.pdf]

Supplementary Information for: “MELD-accelerated Molecular Dynamics help determine amyloid fibril structures”

Bhanita Sharma[1] and Ken A. Dill[1,2,3,*]

[1]Laufer Center for Physical and Quantitative Biology, Stony Brook University, Stony Brook, NY, 11794
[2]Department of Physics and Astronomy, Stony Brook University, Stony Brook, NY 11794
[3]Departments of Chemistry and Physics, Stony Brook University, Stony Brook, NY, 11794
[*]E-mail: dill@laufercenter.org

July 30, 2021

Selection of fibrils for MELD x MD simulation

From the database of 109 structures of Stanković et. al for amyloid fibrils, we have selected 12 fibrils for short-peptides, which represents different classes of steric zipper. The structure of these fibrils are solved by X-ray diffraction. Classes 1–4 have parallel alignment of β-strands and classes 5–8 have antiparallel arrangement of β-strands within each β-sheets. The fibril structures for long fibrils are selected considering various parameters of monomeric sequence length, number of oligomeric chains in the fibrils, and total fibril length. We consider the peptide sequence length higher than 10 residues as long fibrils. The monomeric sequence length of the peptide monomers of these fibrils varies from 11(PDB 2m5n) to 79(PDB 2kj3) residues. The oligomeric state of these fibrils are also different (trimer for PDB 2kj3, to 16-mer for PDB 2m5n). The total fibril sequence lengths for PDB 2beg, 2e8d and 2m5n is below 200, whereas PDB 2lnq, 2kj3 and 2mxu are longer. The structure of PDB 2beg is solved by solution NMR, while all other structures by solid state NMR.

Short fibril Systems (peptides smaller than 10 resides)

PDB ID	UniprotKB	Name	Experiment	Steric Zipper class
2omq	P01308	Human insulin	X-ray Diffraction	class 7
2ona	-	Aβ(35-40)	X-ray Diffraction	class 8
2onv	-	Aβ(37-42)	X-ray Diffraction	class 4
3fva	P67986	Elk prion	X-ray Diffraction	class 1
3loz	P61769	β2-microglobulin	X-ray Diffraction	class 5
3nhc	P04156	Human prion protein (127-132)	X-ray Diffraction	class 8
3nve	P04273	Syrian hamster prion (138-143)	X-ray Diffraction	class 6
3ovl	P10636	Tau	X-ray Diffraction	class 1
3ow9	P05067	Aβ(16-21)	X-ray Diffraction	class 5
3ppd	P15309	Prostatic Acid Phosphatase	X-ray Diffraction	class 1
4onk	-	[Leu-5]-Enkephalin mutant	X-ray Diffraction	class 6
4r0p	P61626	Human lysozyme C (46-61)	X-ray Diffraction	class 1

Long fibril Systems

PDB ID	UniprotKB	Name	Experiment	Computation
2beg	P05067	Aβ(1-42)	NMR	CYANA
2e8d	P61769	β2-microglobulin	SSNMR	CNS, SpinSight, Felix
2kj3	Q03689	HET-s(218-289) prion	SSNMR	CYANA, CNS
2lnq	P05067	Aβ(15-40) Iowa mutant	SSNMR	XPLOR-NIH
2mxu	P05067	Aβ(1-42)	SSNMR	CYANA, AMBER
2m5n	P02766	Transthyretin(105-115)	SSNMR	CNSSOLVE

Table 1: Our selection of short fibril systems and long fibril systems. The structures for short fibrils are available in the PDB as the file called Biological Assemblies. The long fibrils are experimental PDB structures.

Restraint protocol for short fibrils

Inter-strand distances of 4.8 *Å* are incorporated between Cα atoms of all corresponding residues. Also dihedral angle restraints for the peptide backbone with approximate dihedrals for parallel (phi = -119^{0}, psi = $+113^{0}$) and antiparallel (phi = -139^{0}, psi = $+135^{0}$) are imposed to all residues, with a standard deviation of ± 5 *Å*. Inter-sheet distances of 10 *Å* are incorporated between Cα atoms of three central residues of each strand with one another. We arbitrarily decided to enforce all restraints at 80 % accuracy since our restraints data are ambiguous and uncertain.

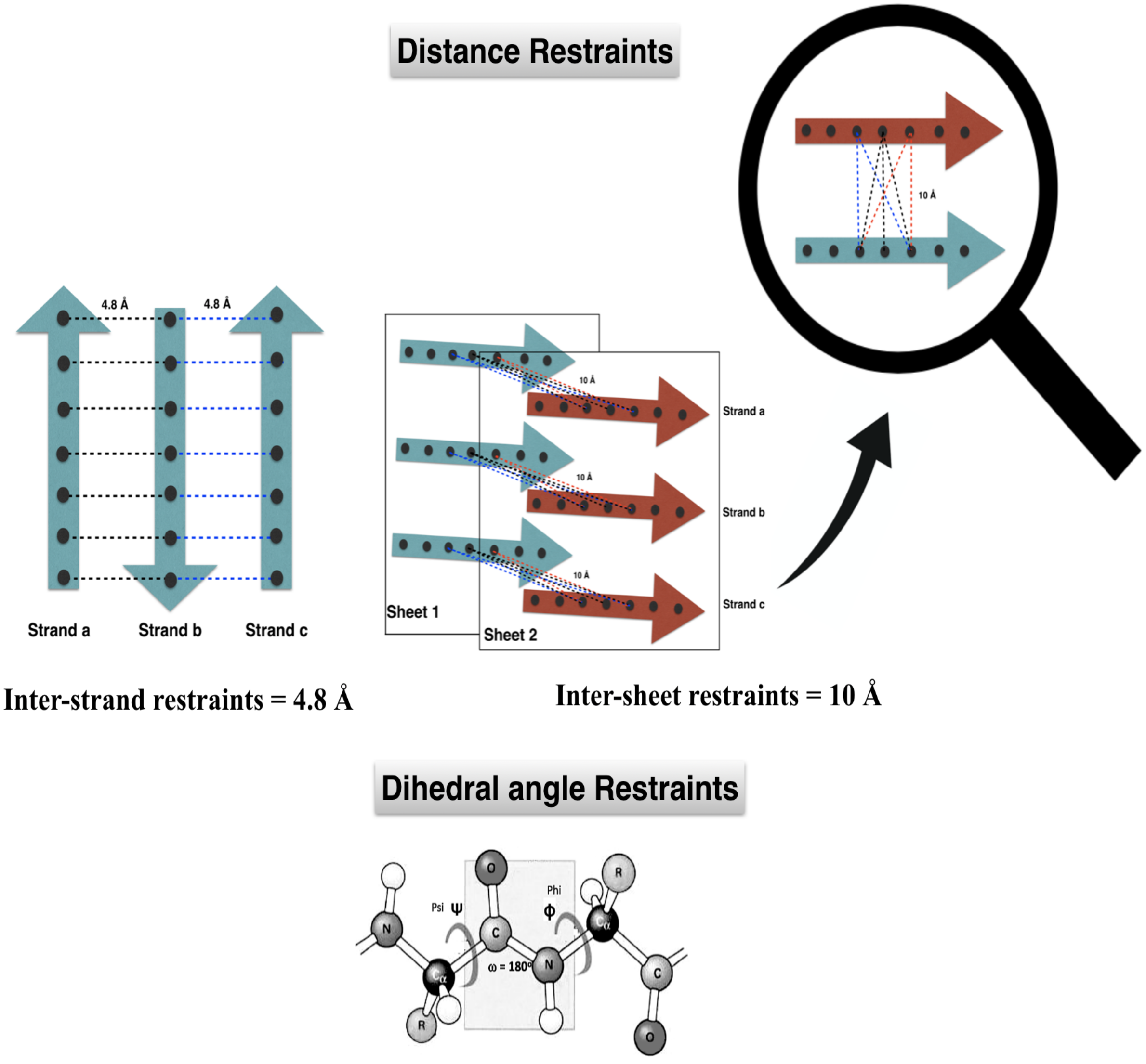

Figure 1: Restraints protocol for short-fibrils.

Restraints for long fibrils

The restraints data (distance restraints and dihedral angle restraints) for long fibrils are obtained from 'NMR Restraints' file of PDB. We enforce atom-atom NMR distance restraints at 80 %. The backbone dihedral angle restraints are also enforced at 80%. The pairwise contacts of the distance restraints used for different fibrils are shown here. The x-axes are scaled to show the fibril size. The experimental structures are shown on the right, with distance restraints data (red) used in the study.

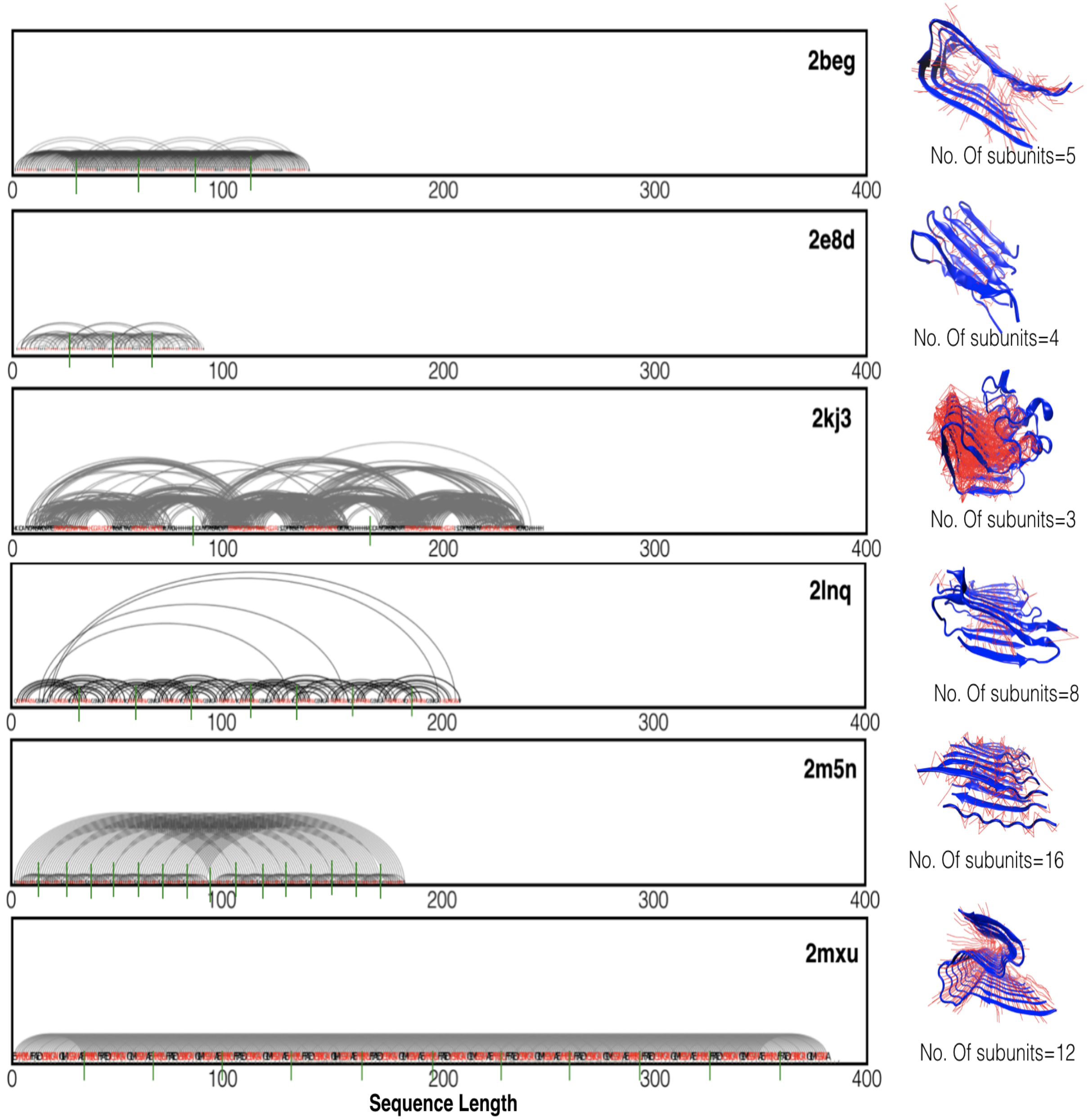

Figure 2: Distance restraints data used in MELD simulations for long fibrils.

MELD x MD simulation for the conversion of extended monomers to trimer

The protocol for generating fibril structure for longer-chain fibrils is to first generate trimeric structure starting from the extended monomer chains. We then build up the actual fibril from the trimeric structure (except for PDB 2kj3). For PDB 2kj3, first the monomer structure is generated from the extended chain, and then fibril structure is build up. The RMSD vs. time plots and RMSD histograms for the conversion of monomers to trimer are shown below. The errorbar is shown at 5.0 $\AA$, the cutoff for folded to native fibril. The RMSD data are for the five lowest temperature replicas, the same replicas that were clustered for analysis.

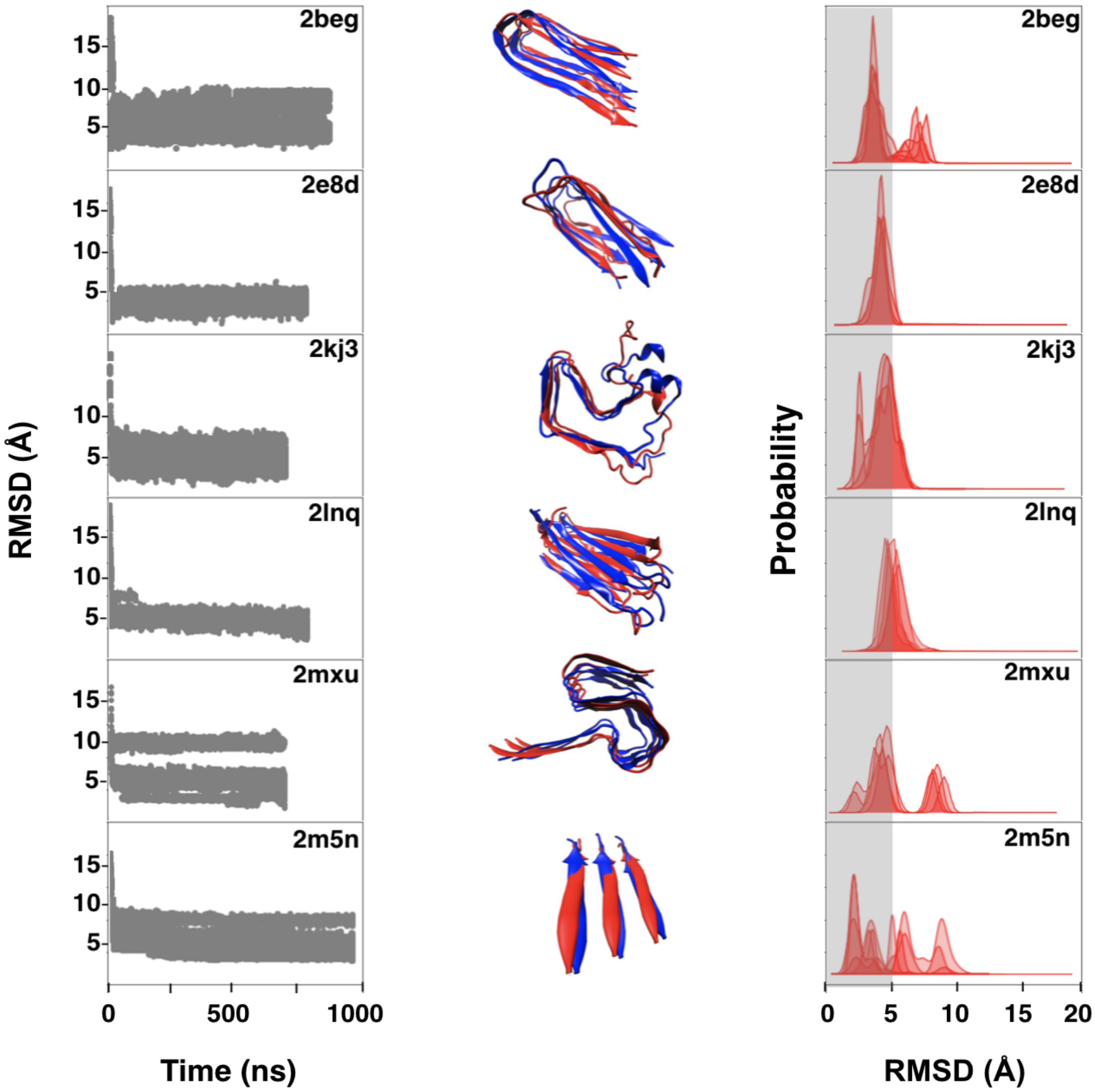

Figure 3: (left) RMSD vs. time and (right) RMSD histogram for the conversion of extended monomers to trimer. For PDB 2kj3, we generated monomer structure from extended chain.

MELD x MD simulation with limited information

For both short and long fibrils, we have simulated a set of simulations with limited restraints data.

(a) The restraints protocols used for short fibrils using limited information are as follows:

1. MELD without dihedral angle restraint (S1): Here we removed the dihedral angle restraints information, but keeping the distance restraints intact. These simulations resulted in fibril structures similar to the general protocol, and removing dihedral angle restraints did not change the population of the top cluster in most cases. However, the peptide backbones are more flexible compared to the general protocol.

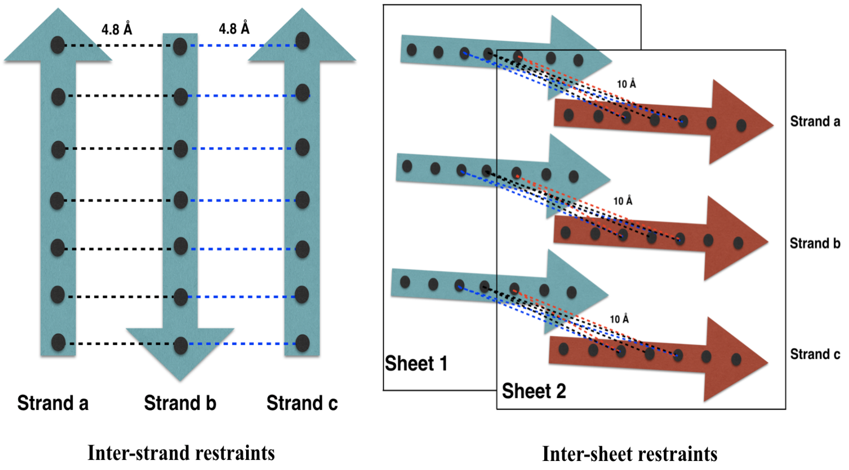

Figure 4: Restraints protocol for system S1.

2. MELD without dihedral angle and limited distance restraints (S2) : Here we limited the number of distance restraints used, but kept the information of strand-arrangements (parallel/anti-parallel) intact. For 7 out of 12 cases, the most populated cluster is within 5.0 *Å* RMSD from reference PDB.

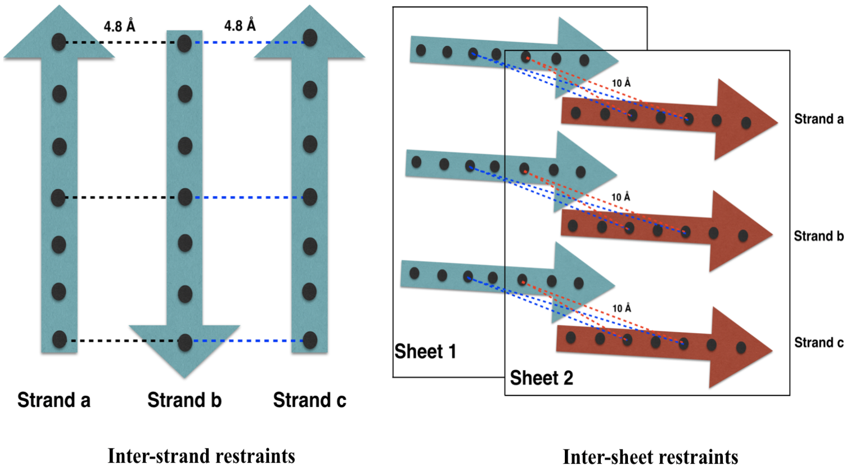

Figure 5: Restraints protocol for system S2.

3. Unguided MELD Simulations (S3): At last we asked if the physical computations alone, without specific information, could predict arrangement of strands in short fibrils. A set of simulations were run without any informative distance restraints and dihedral angle information. However, to keep the peptide monomers together and to avoid straying too far from each other, we used one distance restrain of 4.8 $\AA$ between the Cα atoms of the central residues of each peptide strand. We call it 'unguided' because here we have not used any directive restraints. The top MELD cluster of these unguided simulations are far from the reference PDB.

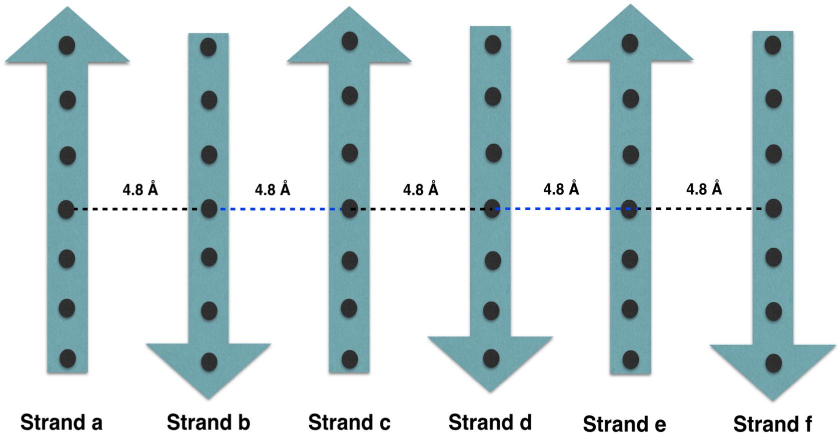

Figure 6: Restraints protocol for system S3.

Supplementary Figures 4-6 represents the restraints protocols for systems S1, S2 and S3. **Supplementary Table 4** shows the amount of restraints used in these different systems. The RMSD vs. time and RMSD histograms for system S2 and S3 are shown in **Supplementary Figures 7-8**. System S1 is omitted, as the population distribution are similar to the general protocol. The results of all the different systems are summarised in **Supplementary Table 2**.

(b) To assess the quality of MELD x MD structure prediction with limited restraints information for long fibrils, we simulated a set of systems for four structures, namely 2beg, 2e8d, 2mxu and 2m5n. We skipped PDB 2lnq and 2kj3 for limited restraints analysis. PBD 2kj3 with monomer chain-length of 79 amino acid is the longest in our selection of fibrils; and form cross-β-sheets with residues within the individual chains. Due to its large size, we assumed that simulations without informative restraints would not be successful. Whereas MELD prediction for 2lnq was unsuccessful even with SSNMR restraints, as restraints per residue is lowest for 2lnq.

The restraint protocol used for these different simulations are :

System L1: Here we simulated the structures with NMR distance restraints, but without any dihedral angle restraints information. The most populated clusters for all cases are within 5.0 $\AA$ RMSD from reference PDB.

System L2: Next, we simulated these fibril structures using restraints protocol similar to our MELD simulation of short fibrils. PDB 2m5n has two straight cross-β sheets similar to short fibrils. Therefore, the restraints protocol is also exactly similar. Fibrils of PDB 2beg and 2e8d have turns or loop regions between strands. These structures are characterised by U-shaped structure, with strand-loop-strand (β-loop-β) arrangement. Considering this knowledge as external information, we incorporated intra-sheet distance restraint of 4.8 $\AA$ between residues of the monomers. We used dihedral angle restraint for parallel β-sheets (phi=-119, psi= +113) for all residues except the residues in the turn regions. We also applied some intra-monomer distance restraints (similar to inter-sheet distance restraints for short fibril) of 10 Å between Cα atoms of residues of one end of each fibril monomer to the other end. PDB 2mxu is an S-shape fibril. However, we used the same protocol of restraints as for the U-shaped structure. The reason here is, defining a general restraints protocol for S-shaped fibril is difficult without the availability of proper experimental data. We observed that even with these limited pieces of information, the centroid of the most populous clusters are within 4.0 $\AA$ RMSD from the reference fibril structure, except for PDB 2mxu **(Supplementary Figures 9-12)**. The failure of 2mxu is due to enforcing large number of inaccurate restraints at 80% precision, as described in our protocol. Therefore, the MELD prediction is far away from the native fibril structure. These results suggests that although extensive experimental data may not be necessary, however, some limited but qualitatively informative information are required to predict fibril structure.

System L3: We also performed another set of MELD x MD simulations, with the only information of parallel strand arrangement. The restraints input here is the inter-monomer distance restraints of 4.8 $\AA$ between the Cα atoms of the corresponding residues of parallel strands. We did not apply any intramolecular restraints and dihedral angle restraints information. However, we observed that in absence of any intra-monomer restraints in system L3, the RMSD deviations are much higher in all cases. **Supplementary Table 5** shows the amount of restraints used in different systems for long fibrils. The results of all the predicted structures of these different systems are shown in **Supplementary Figures 9-12** and **Supplementary Table 3**.

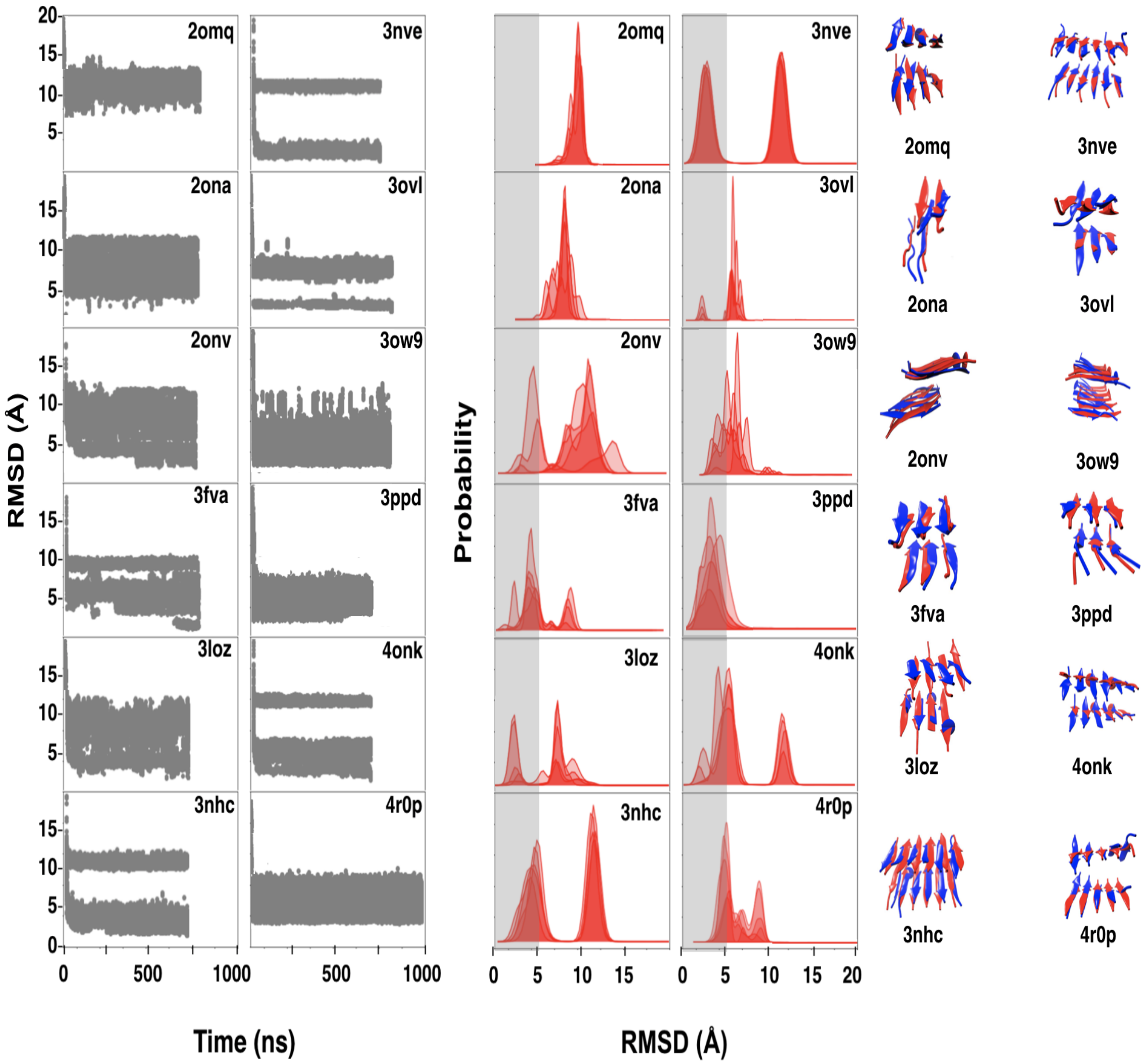

Figure 7: (left) RMSD vs. time and (right) RMSD histograms for system S2. While more restraints naturally generate correct predictions, limited restraints can still generate correct structures in most cases.

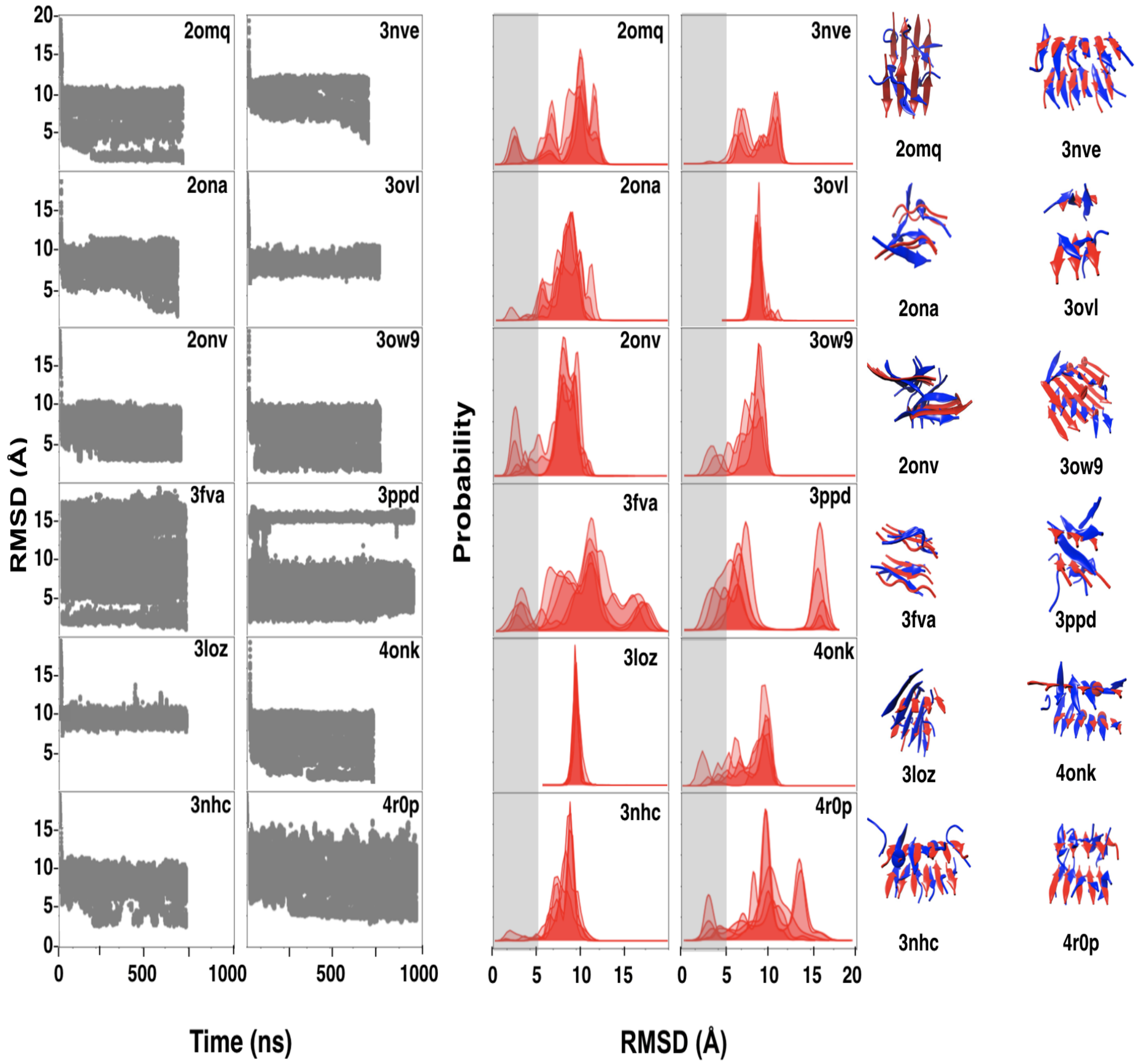

Figure 8: (left) RMSD vs. time and (right) RMSD histograms for system S3. The unguided MELD simulations of system S3 generates oligomeric structures in all cases, and for 8 cases out of 12, the most populated cluster in MELD gives structures with correct strand arrangements (parallel/anti-parallel) as of the reference PDB structure. For other 4 fibrils, the oligomeric structures are found to be a random mix of both parallel and anti-parallel strands. However, the RMSDs are higher in all cases.

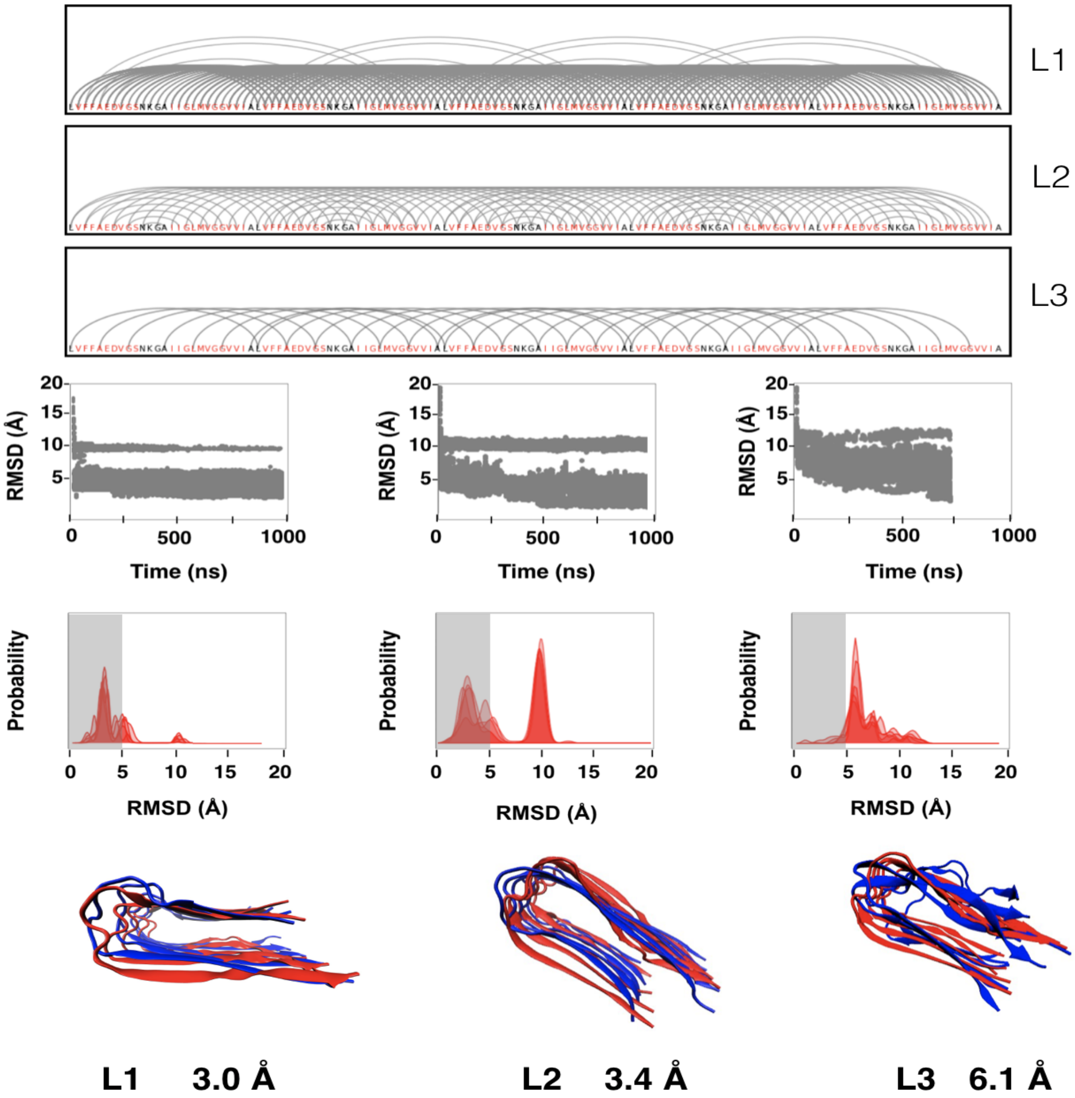

Figure 9: The pairwise contacts, RMSD vs. time and RMSD histograms are plotted for PDB 2beg in different simulations.

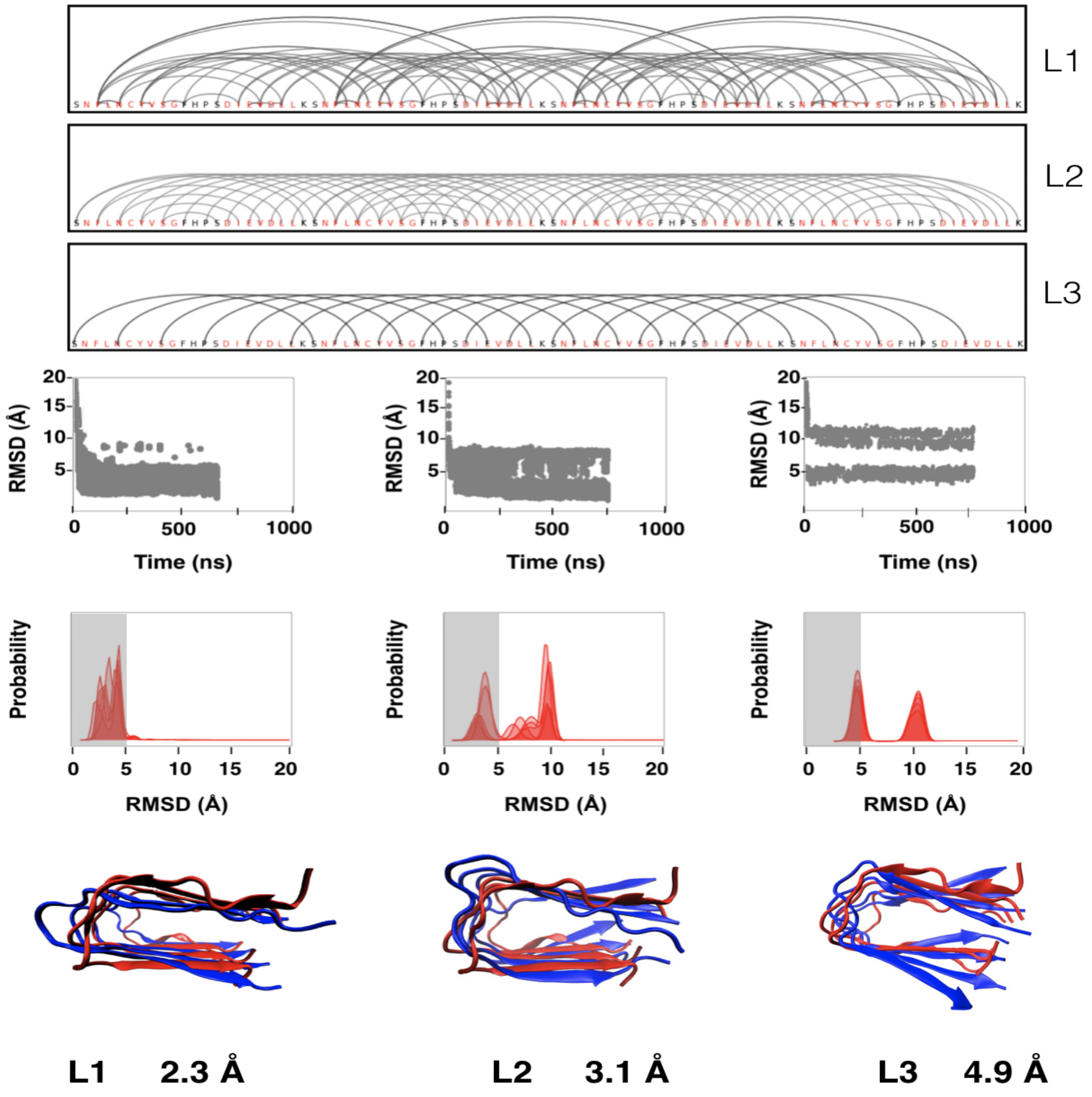

Figure 10: The pairwise contacts, RMSD vs. time and RMSD histograms plotted for PDB 2e8d in different simulations.

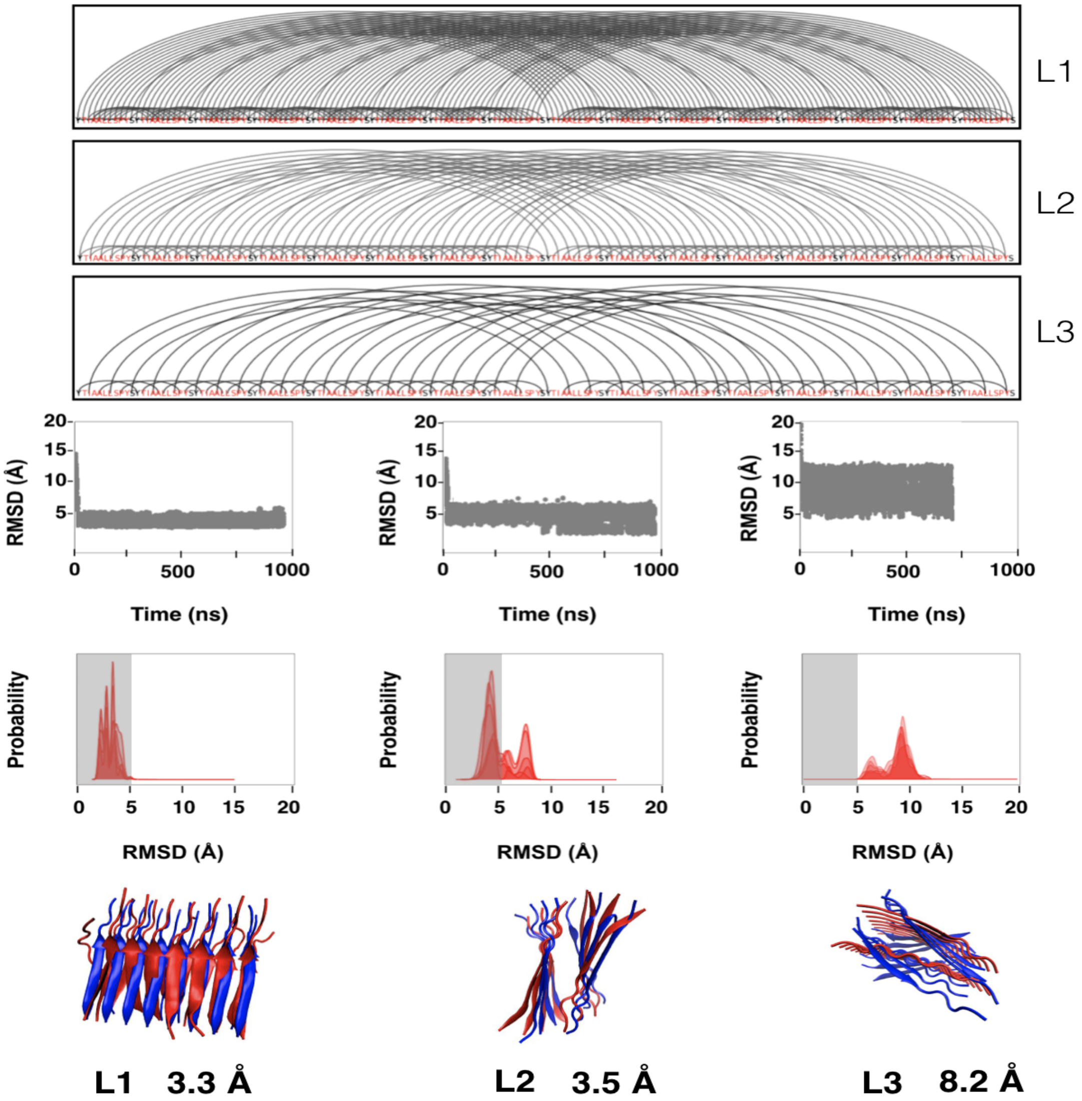

Figure 11: The pairwise contacts, RMSD vs. time and RMSD histograms plotted for PDB 2m5n in different simulations.

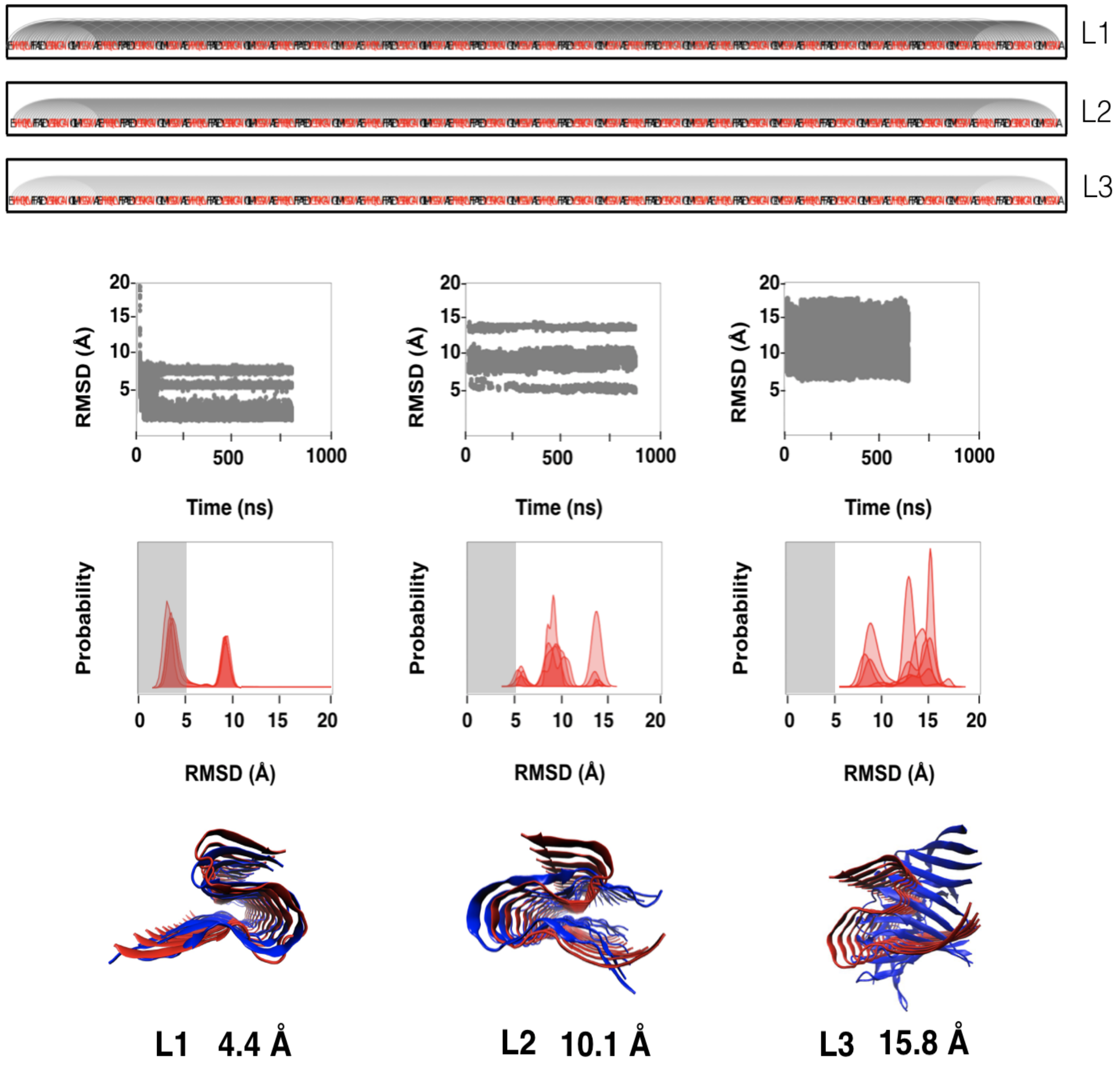

Figure 12: The pairwise contacts, RMSD vs. time and RMSD histograms plotted for PDB 2mxu in different simulations. The most populated cluster for system L1 is within 5.0 $\AA$ RMSD from the reference PDB. For system L2, the MELD prediction is far away from the native fibril structure. PDB 2mxu is an S-shaped fibril, whereas our general protocol of restraints is for U-shaped structure; and these large number of inaccurate restraints were enforced at 80% precision. Whereas in absence of any intra-monomer restraints in system L3, the RMSD deviation is much higher.

Systems S1

PDB ID	RMSD (Å)			Population		
	c0	c1	c2	c0	c1	c2
2omq	7.6	8.2	**6.0**	0.3	0.2	0.1
2ona	7.0	**5.0**	5.5	0.4	0.1	0.1
2onv	**3.8**	4.5	4.4	0.4	0.2	0.1
3fva	**2.0**	5.6	9.5	0.5	0.3	0.1
3loz	**2.5**	8.5	5.4	0.3	0.3	0.1
3nhc	**2.2**	7.6	2.5	0.3	0.2	0.1
3nve	**2.6**	11.8	11.3	0.6	0.2	0.1
3ovl	**3.4**	8.2	3.4	0.5	0.2	0.1
3ow9	**2.2**	9.8	2.6	0.4	0.3	0.1
3ppd	**1.5**	8.5	3.6	0.4	0.3	0.1
4onk	**3.8**	5.4	5.8	0.5	0.3	0.1
4r0p	**2.9**	10.5	3.4	0.4	0.4	0.01

Systems S2

PDB ID	RMSD (Å)			Population		
	c0	c1	c2	c0	c1	c2
2omq	10.0	7.8	**6.5**	0.3	0.1	0.1
2ona	7.9	8.2	**6.0**	0.4	0.1	0.1
2onv	12.2	**4.0**	8.2	0.2	0.1	0.1
3fva	4.5	8.2	**3.0**	0.4	0.1	0.1
3loz	7.5	**2.5**	8.8	0.3	0.2	0.1
3nhc	**4.0**	12.2	11.5	0.4	0.1	0.1
3nve	**2.5**	12.1	11.2	0.5	0.2	0.1
3ovl	6.1	7.2	**2.6**	0.5	0.2	0.5
3ow9	4.8	7.4	**3.2**	0.6	0.1	0.1
3ppd	**2.5**	8.6	3.0	0.4	0.2	0.1
4onk	5.0	12.1	**4.6**	0.4	0.2	0.1
4r0p	**4.8**	8.8	6.9	0.5	0.2	0.1

Systems S3

PDB ID	RMSD (Å)			Population		
	c0	c1	c2	c0	c1	c2
2omq	10.2	6.0	**3.2**	0.2	0.1	0.1
2ona	8.9	7.4	**5.6**	0.3	0.1	0.1
2onv	**7.5**	9.2	8.3	0.2	0.1	0.1
3fva	12.1	9.3	**5.8**	0.1	0.1	0.1
3loz	9.8	**8.8**	10.6	0.8	0.1	0.1
3nhc	**7.2**	8.6	9.2	0.2	0.2	0.1
3nve	**7.0**	11.2	8.6	0.3	0.2	0.1
3ovl	8.6	**7.4**	9.3	0.7	0.1	0.1
3ow9	8.8	7.2	**4.0**	0.6	0.2	0.1
3ppd	7.2	**5.3**	14.1	0.2	0.1	0.1
4onk	9.1	7.0	**4.6**	0.2	0.1	0.1
4r0p	9.6	13.1	**7.8**	0.1	0.1	0.1

Table 2: MELD x MD results with RMSD to PDB reference and cluster populations for the top three clusters (c0-c2) for short fibril systems with limited information.

Systems L1

PDB ID	RMSD (Å)			Population		
	c0	c1	c2	c0	c1	c2
2beg	**3.0**	5.2	2.7	0.5	0.3	0.1
2e8d	**2.3**	3.2	4.2	0.3	0.2	0.1
2mxu	4.4	8.2	**3.8**	0.4	0.3	0.1
2m5n	3.3	**2.8**	4.0	0.4	0.2	0.1

Systems L2

	c0	c1	c2	c0	c1	c2
2beg	**3.4**	10.1	4.4	0.5	0.3	0.1
2e8d	**3.1**	10.3	3.6	0.4	0.3	0.1
2mxu	10.1	**5.6**	13.1	0.3	0.2	0.1
2m5n	**3.5**	8.2	4.4	0.5	0.3	0.1

Systems L3

	c0	c1	c2	c0	c1	c2
2beg	6.1	11.2	**5.4**	0.4	0.3	0.2
2e8d	**4.9**	10.8	5.3	0.4	0.2	0.1
2mxu	15.8	**8.9**	13.5	0.2	0.1	0.1
2m5n	8.2	**5.3**	6.8	0.3	0.2	0.1

Table 3: MELD x MD results with RMSD to PDB reference and cluster populations for the top three clusters (c0-c2) for long fibril systems with limited information.

System S1

Fibrils	Distance Restraints				Dihedral restraints		RMSD (Å)
PDB ID	Total	Intramonomer	Intermonomer	Restraints per amino acid	ϕ	ψ	c0
2omq	44	0	44	0.9	0	0	7.6
2ona	60	0	60	1.3	0	0	7.0
2onv	28	0	28	1.2	0	0	3.8
3fva	42	0	42	1.2	0	0	2.0
3loz	48	0	48	1.0	0	0	2.5
3nhc	96	0	96	1.3	0	0	2.2
3nve	94	0	94	1.3	0	0	2.6
3ovl	46	0	46	0.9	0	0	3.4
3ow9	72	0	72	1.0	0	0	2.2
3ppd	42	0	42	1.2	0	0	1.5
4onk	62	0	62	1.0	0	0	3.8
4r0p	78	0	78	1.3	0	0	2.9
System S2							
2omq	22	0	22	0.5	0	0	10.0
2ona	24	0	24	0.5	0	0	7.9
2onv	12	0	12	0.5	0	0	12.2
3fva	28	0	28	0.8	0	0	4.5
3loz	22	0	22	0.5	0	0	7.5
3nhc	64	0	64	0.9	0	0	4.0
3nve	42	0	42	0.6	0	0	2.5
3ovl	22	0	22	0.5	0	0	6.1
3ow9	32	0	32	0.4	0	0	4.8
3ppd	28	0	28	0.8	0	0	2.5
4onk	32	0	32	0.5	0	0	5.0
4r0p	26	0	26	0.4	0	0	4.8
System S3							
2omq	10	0	10	0.2	0	0	10.2
2ona	10	0	10	0.2	0	0	8.9
2onv	4	0	4	0.2	0	0	7.5
3fva	7	0	7	0.2	0	0	12.1
3loz	10	0	10	0.2	0	0	9.8
3nhc	32	0	32	0.4	0	0	7.2
3nve	22	0	22	0.3	0	0	7.0
3ovl	14	0	14	0.3	0	0	8.6
3ow9	16	0	16	0.2	0	0	8.8
3ppd	7	0	7	0.2	0	0	7.2
4onk	16	0	16	0.3	0	0	9.1
4r0p	13	0	13	0.2	0	0	9.6

Table 4: Limited restraints information used in different systems for short fibrils. In System S1 dihedral angle restrains are removed. In S2, distance restraints are limited. In S3, only a single set of distance restraints between central residues of each peptide strand are imposed.

System L0

Fibrils	Distance Restraints				Dihedral restraints		RMSD (Å)
PDB ID	Total	Intramonomer	Intermonomer	Restraints/residue	ϕ	ψ	c0
2beg	698	170	528	7.2	125	125	2.5
2e8d	115	52	63	3.1	80	80	2.0
2kj3	5975	5552	423	26.2	123	123	3.2
2lnq	271	88	183	2.5	128	128	5.2
2mxu	8211	4967	3244	23.1	336	336	3.4
2m5n	868	560	126	6.8	160	160	3.0
System L1							
2beg	698	170	528	5.3	0	0	3.0
2e8d	115	52	63	1.3	0	0	2.3
2m5n	868	560	126	4.9	0	0	3.3
2mxu	8211	4967	3244	21.4	0	0	4.4
System L2							
2beg	184	80	104	1.4	0	0	3.4
2e8d	106	40	66	1.2	0	0	3.1
2m5n	242	0	242	1.4	0	0	3.5
2mxu	452	100	352	1.2	0	0	10.1
System L3							
2beg	104	0	104	0.8	0	0	6.1
2e8d	66	0	66	0.7	0	0	4.9
2m5n	100	0	100	0.6	0	0	8.2
2mxu	352	0	352	0.9	0	0	15.8

Table 5: Limited restraints information used in different systems for long fibrils. System L0 is when all NMR restraints are imposed. In system L1, dihedral angle restraints are removed. In system L2, the restraints protocol is similar to short fibrils. In system L3, only intermonomer distance restraints of 4.8 *Å* for parallel strands are imposed. System L0 is a reference for, when all NMR restraints are imposed. Dihedral angle restraints are also considered in calculating restraints per residue.

The failure modes, and recovering from them

In order to recover the failed MELD structures, we carried out another set of simulations. Our general MELD protocol for short fibrils failed to predict structures for two short fibrils 2omq and 2ona. To improve the structure prediction, we have added distance restraints with accurate inter-sheet distances, derived from the reference PDB. The restraints are applied between Cα atoms of all corresponding residues of the two sheets to ensure a similar pattern of steric-zipper interface as in the PDB. A total of 24 accurate inter-sheet distance restraints are added in both caese. On the other hand the failure of PDB 2lnq is mostly associated with the intrinsic flexibility of the peptide in simulations, as the number of inter-monomer β-sheet alignment restraints were not sufficient. Therefore we have added an extra 60 inter-strand distance restraints between residues according to the reference PDB structure. These inter-strand distance restraints of 4.8 $\AA$ are added between Cα atoms of all corresponding residues.

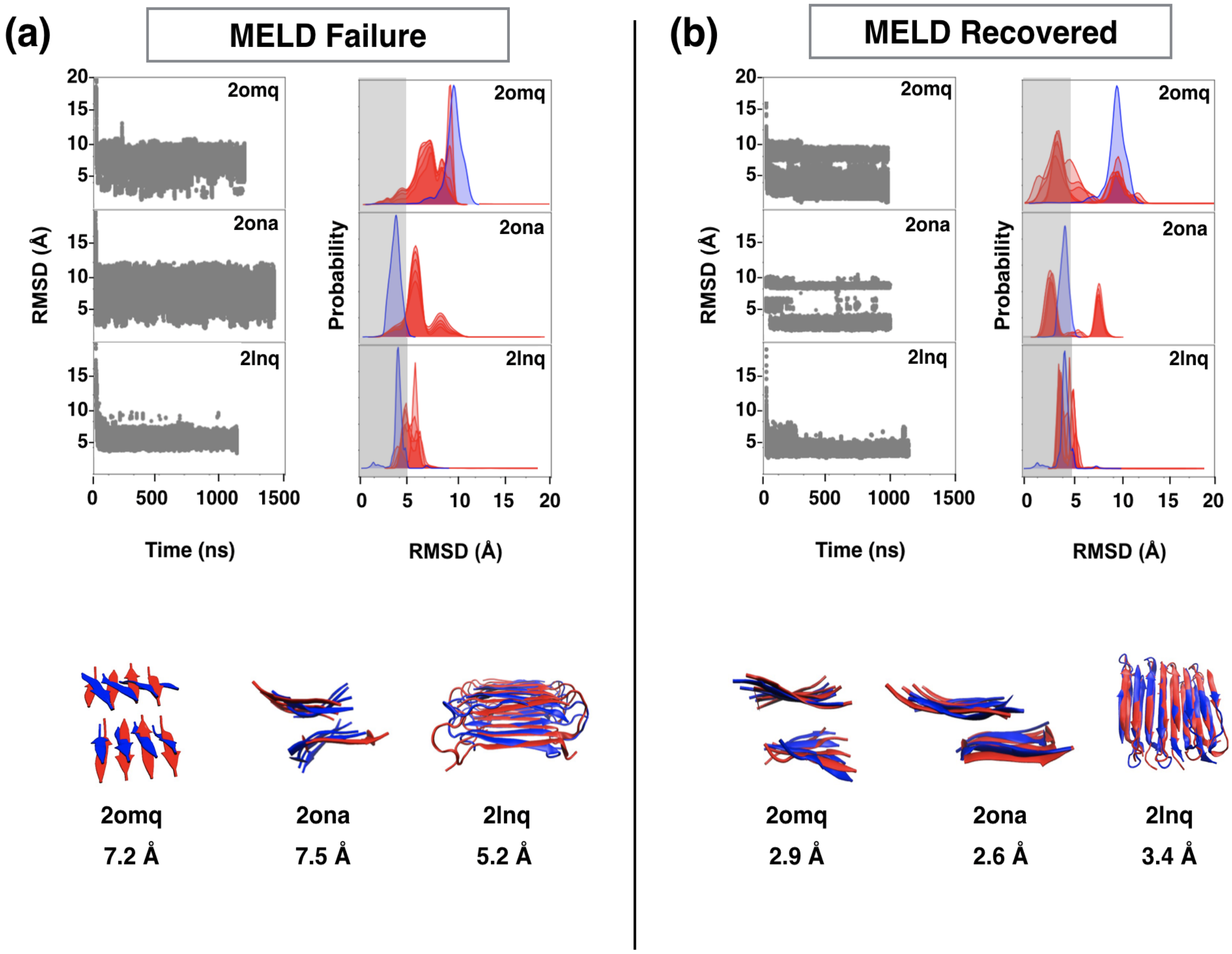

Figure 13: Sampled distributions to identify source of errors. MELD x MD distributions (shown in red distribution) compared to MD sampling distribution (shown in blue distribution) around the PDB native structures. (a) MELD failures. (b) Recovery of the failed structures in MELD. The MELD predicted structure (blue) vs. reference PDB (red) are shown at the bottom.

The replica exchange condition of different MELD x MD simulations

High temperature replicas (bigger replica indices) mixed well with the low temperature ones. The exchanges are attempted every 50 ps, and the acceptance probability of replica exchanges are typically at about 30-50 %.

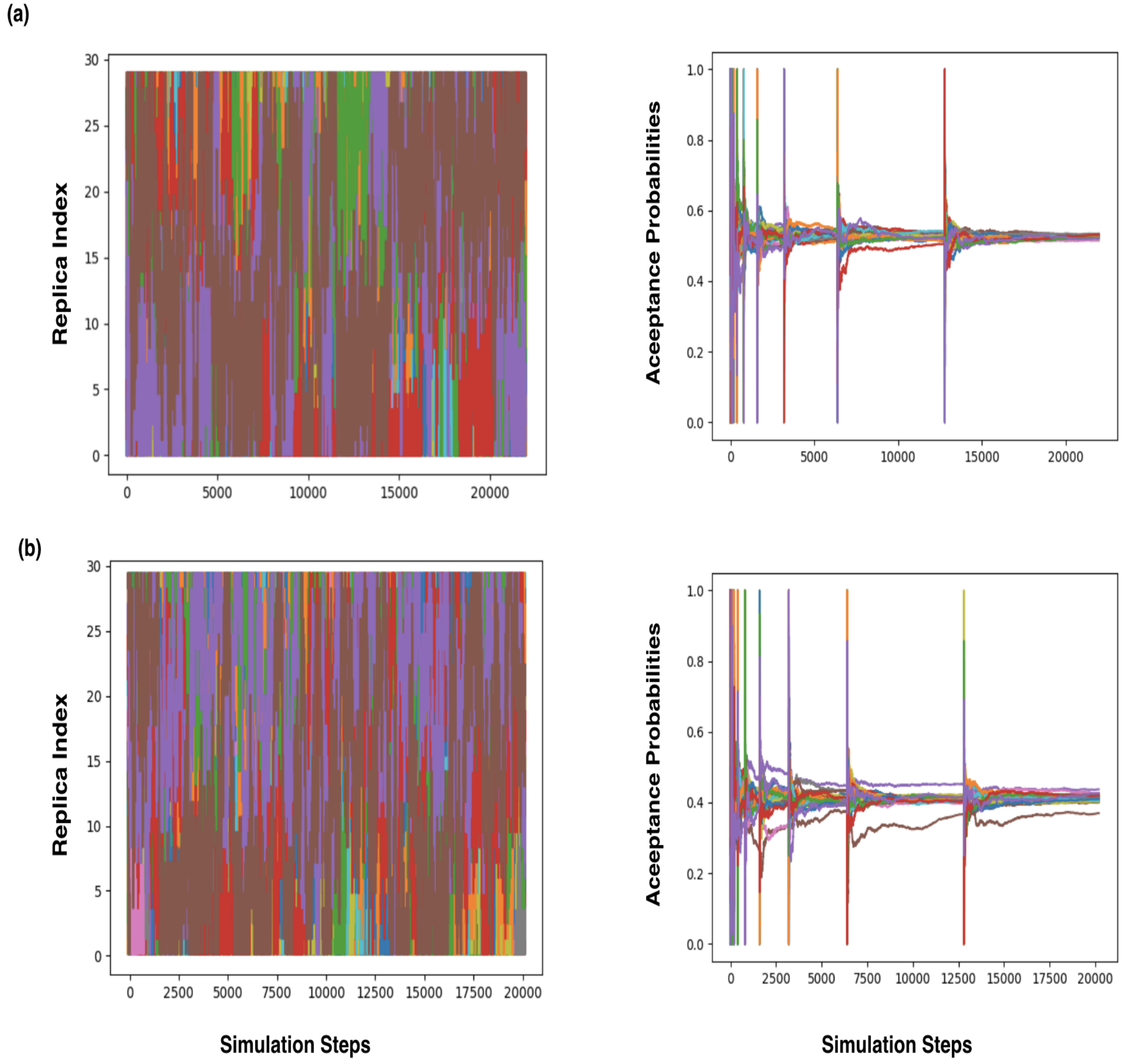

Figure 14: The replica exchange conditions of (a) 3nhc, short fibril and (b) 2e8d, long fibril are shown as an example.The left panel shows the exchanges among replicas over simulation time. The right panel shows the acceptance probability over simulation time.

In some cases we have observed poor replica exchanges for simulations of long fibrils starting from extended monomers to form fibril structure. The poor replica exchanges may reduce sampling and limit accurate structure prediction. Increasing the number of replicas improved the exchanges. The exchanges among replicas, and acceptance probability of replica exchanges over simulation

time for PDB 2mxu are shown.

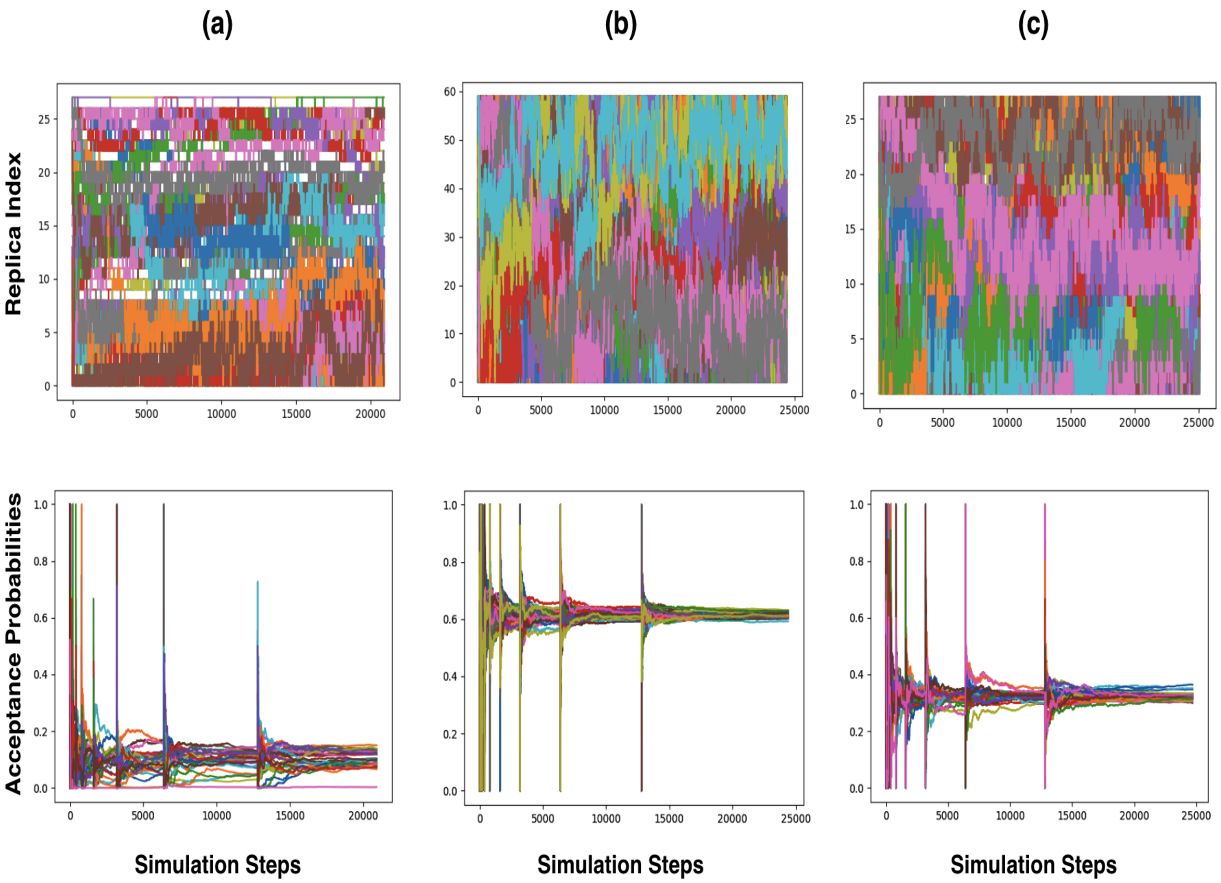

Figure 15: The exchanges among different replicas (above) and acceptance probability (below) of PDB 2mxu in different MELD x MD simulations. (a) shows poor exchange, with 28 replicas while starting from extended monomers as initial configuration to form fibril; (b) shows simulation with 60 replicas where the high temperature replicas (bigger replica indices) mixed well with low temperature ones; whereas in (c) even with 28 replicas, the replica exchange becomes much better, while forming fibril structure starting from trimers.

Convergence of simulations

To check the convergence of our replica exchange simulation, we checked the RMSD histograms of all 30 replicas relative to the last frame of the simulation. Converged simulations would give overlapping histograms. It is observed that in some cases the higher replica index trajectories are not converged.

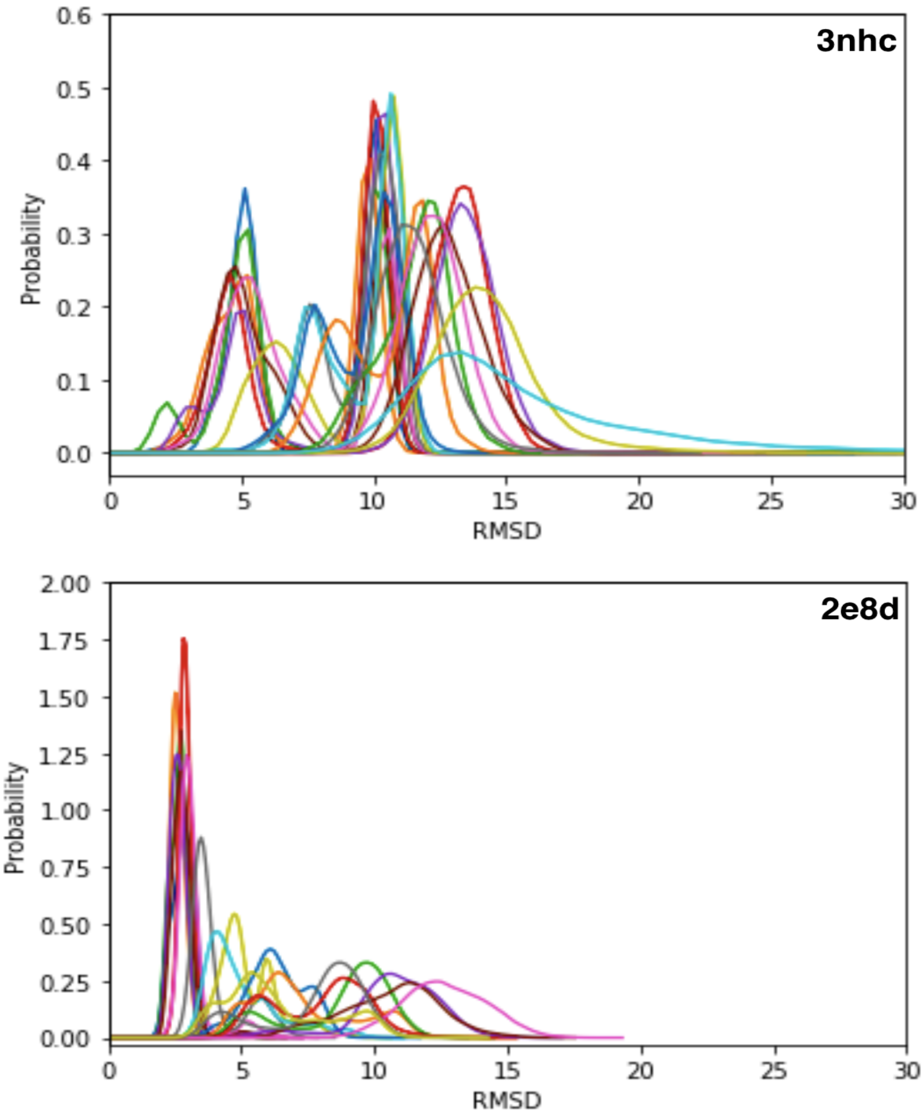

Figure 16: RMSD histograms for (a) 3nhc, short fibril and (b) 2e8d, long fibril are shown as an example, where higher replica index trajectories are not converged.
