# Supplementary material for: MELD-accelerated molecular dynamics help determine amyloid fibril structures
Source: Commun Biol. 2021 Aug 5;4:942. doi: 10.1038/s42003-021-02461-y (PMC8342454; doi:10.1038/s42003-021-02461-y)
Supplement: Supplementary file 3 — Description of Supplementary Files [file 42003_2021_2461_MOESM3_ESM.pdf]

Description of Additional Supplementary Files

File name: Supplementary Data

Description: Data for Figure 1, Figure 3, Figure 5, Figure 6 and Figure 7. Captions are given at the top of each Excel file.
