# Supplementary material for: MELD-accelerated molecular dynamics help determine amyloid fibril structures
Source: Commun Biol. 2021 Aug 5;4:942. doi: 10.1038/s42003-021-02461-y (PMC8342454; doi:10.1038/s42003-021-02461-y)
Supplement: Supplementary file 5 — Reporting Summary [file 42003_2021_2461_MOESM5_ESM.pdf]

nature research

Corresponding author(s): Ken A. Dill

Last updated by author(s): 07/09/2021

Reporting Summary

Nature Research wishes to improve the reproducibility of the work that we publish. This form provides structure for consistency and transparency in reporting. For further information on Nature Research policies, see Authors & Referees and the Editorial Policy Checklist.

Statistics

For all statistical analyses, confirm that the following items are present in the figure legend, table legend, main text, or Methods section.

n/a	Confirmed	
☐	☒	The exact sample size (*n*) for each experimental group/condition, given as a discrete number and unit of measurement
☒	☐	A statement on whether measurements were taken from distinct samples or whether the same sample was measured repeatedly
☒	☐	The statistical test(s) used AND whether they are one- or two-sided<br>*Only common tests should be described solely by name; describe more complex techniques in the Methods section.*
☒	☐	A description of all covariates tested
☒	☐	A description of any assumptions or corrections, such as tests of normality and adjustment for multiple comparisons
☒	☐	A full description of the statistical parameters including central tendency (e.g. means) or other basic estimates (e.g. regression coefficient) AND variation (e.g. standard deviation) or associated estimates of uncertainty (e.g. confidence intervals)
☒	☐	For null hypothesis testing, the test statistic (e.g. *F*, *t*, *r*) with confidence intervals, effect sizes, degrees of freedom and *P* value noted<br>*Give P values as exact values whenever suitable.*
☐	☒	For Bayesian analysis, information on the choice of priors and Markov chain Monte Carlo settings
☐	☒	For hierarchical and complex designs, identification of the appropriate level for tests and full reporting of outcomes
☒	☐	Estimates of effect sizes (e.g. Cohen's *d*, Pearson's *r*), indicating how they were calculated

Our web collection on statistics for biologists contains articles on many of the points above.

Software and code

Policy information about availability of computer code

Data collection	We have used MELD (Modeling Employing Limited Data) that can speed up MD simulations by using external information. The restraints data (distance restraints and dihedral angle restraints) used in MELD for long fibrils are obtained from the 'NMR Restraints' file of reference PDB from the RCSB PDB website.
Data analysis	The results are extracted by clustering the trajectories using average-linkage hierarchical agglomerative algorithm, with a combination of scripts included with MELD x MD, and CPPTRAJ from AmberTools17.

For manuscripts utilizing custom algorithms or software that are central to the research but not yet described in published literature, software must be made available to editors/reviewers. We strongly encourage code deposition in a community repository (e.g. GitHub). See the Nature Research guidelines for submitting code & software for further information.

Data

Policy information about availability of data

All manuscripts must include a data availability statement. This statement should provide the following information, where applicable:

- Accession codes, unique identifiers, or web links for publicly available datasets
- A list of figures that have associated raw data
- A description of any restrictions on data availability

The authors declare that all relevant data supporting the findings of this study are available within the Article file, Supplementary Information file and the Supplementary Data file.

Field-specific reporting

Please select the one below that is the best fit for your research. If you are not sure, read the appropriate sections before making your selection.

☒ Life sciences ☐ Behavioural & social sciences ☐ Ecological, evolutionary & environmental sciences

For a reference copy of the document with all sections, see nature.com/documents/nr-reporting-summary-flat.pdf

Life sciences study design

All studies must disclose on these points even when the disclosure is negative.

Sample size	In order to validate the use of MELD x MD in predicting amyloid fibril structures, we aimed to reproduce the experimentally determined structures of 12 short fibrils and 6 large fibrils, available in the database of 109 amyloid structures of Stanković et. al [reference 25 in the manuscript]. The 12 short fibrils represents different classes of steric zippers. On the other hand, out of the 25 amyloid fibril structures deposited in the PDB that have been elucidated by using SSNMR as the main method, we have selected 5 long PDB structures; and another one structure selected from PDB solved by NMR, as our test sets. The structures are selected considering various parameters of monomeric sequence length, number of oligomeric chains in the fibrils, and total fibril length.
Data exclusions	No data have been excluded from the analyses.
Replication	Our reported results of MELD x MD simulation with different randomly placed initial configurations of peptides show that, in the limit of converged sampling and perfect force field accuracy, using same restraint information yield the same lowest free energy structures, irrespective of the initial structures.
Randomization	No randomization was necessary for this study, because we are comparing the MELD x MD predicted structure to the native fibril structure. Randomization is not generally used in this field.
Blinding	Since this study is a test to reproduce the experimentally determined structures of amyloid fibrils using MELD x MD, therefore, blinding is not relevant to the study. Blinding during analysis is not feasible as calculating RMSD errors with the reference PDB structure is what determines the success/failures of our protocol in our analysis.

Reporting for specific materials, systems and methods

We require information from authors about some types of materials, experimental systems and methods used in many studies. Here, indicate whether each material, system or method listed is relevant to your study. If you are not sure if a list item applies to your research, read the appropriate section before selecting a response.

Materials & experimental systems

n/a	Involved in the study	
☒	☐	Antibodies
☒	☐	Eukaryotic cell lines
☒	☐	Palaeontology
☒	☐	Animals and other organisms
☒	☐	Human research participants
☒	☐	Clinical data

Methods

n/a	Involved in the study	
☒	☐	ChIP-seq
☒	☐	Flow cytometry
☒	☐	MRI-based neuroimaging
